# Oral Bioactive Self-Nanoemulsifying Drug Delivery Systems of Remdesivir and Baricitinib: A Paradigmatic Case of Drug Repositioning for Cancer Management

**DOI:** 10.3390/molecules28052237

**Published:** 2023-02-28

**Authors:** Mohsin Kazi, Yousef Alanazi, Ashok Kumar, Ahmad Abdul-Wahhab Shahba, Syed Rizwan Ahamad, Khalid M. Alghamdi

**Affiliations:** 1 Department of Pharmaceutics, College of Pharmacy, King Saud University, Riyadh 11451, Saudi Arabia; 2 Vitiligo Research Chair, Department of Dermatology, College of Medicine, King Saud University, Riyadh 11451, Saudi Arabia; 3 Kayyali Research Chair for Pharmaceutical Industries, College of Pharmacy, King Saud University, Riyadh 11451, Saudi Arabia; 4 Department of Pharmaceutical Chemistry, College of Pharmacy, King Saud University, Riyadh 11451, Saudi Arabia; 5 Department of Dermatology, College of Medicine, King Saud University, Riyadh 11451, Saudi Arabia

**Keywords:** natural oils, bioactive self-nanoemulsifying drug delivery systems (bio-SNEDDS), MDA-MB-231 breast cancer cells, A549 lung cancer cells, cytotoxicity

## Abstract

Oral anticancer therapy mostly faces the challenges of low aqueous solubility, poor and irregular absorption from the gastrointestinal tract, food-influenced absorption, high first-pass metabolism, non-targeted delivery, and severe systemic and local adverse effects. Interest has been growing in bioactive self-nanoemulsifying drug delivery systems (bio-SNEDDSs) using lipid-based excipients within nanomedicine. This study aimed to develop novel bio-SNEDDS to deliver antiviral remdesivir and baricitinib for the treatment of breast and lung cancers. Pure natural oils used in bio-SNEDDS were analyzed using GC-MS to examine bioactive constituents. The initial evaluation of bio-SNEDDSs were performed based on self-emulsification assessment, particle size analysis, zeta potential, viscosity measurement, and transmission electron microscopy (TEM). The single and combined anticancer effects of remdesivir and baricitinib in different bio-SNEDDS formulations were investigated in MDA-MB-231 (breast cancer) and A549 (lung cancer) cell lines. The results from the GC-MS analysis of bioactive oils BSO and FSO showed pharmacologically active constituents, such as thymoquinone, isoborneol, paeonol and p-cymenene, and squalene, respectively. The representative F5 bio-SNEDDSs showed relatively uniform, nanosized (247 nm) droplet along with acceptable zeta potential values (+29 mV). The viscosity of the F5 bio-SNEDDS was recorded within 0.69 Cp. The TEM suggested uniform spherical droplets upon aqueous dispersions. Drug-free, remdesivir and baricitinib-loaded bio-SNEDDSs (combined) showed superior anticancer effects with IC50 value that ranged from 1.9–4.2 µg/mL (for breast cancer), 2.4–5.8 µg/mL (for lung cancer), and 3.05–5.44 µg/mL (human fibroblasts cell line). In conclusion, the representative F5 bio-SNEDDS could be a promising candidate for improving the anticancer effect of remdesivir and baricitinib along with their existing antiviral performance in combined dosage form.

## 1. Introduction

Cancer is a multifactorial disease with many aspects influencing its initiation and progression and remains the primary cause of mortality around the globe [1,2]. A significant aspect of developing effective anticancer drugs in humans is to better understand human cancer biology during the preclinical stage of drug discovery [3]. The time-tested hallmarks and enabling factors have provided a useful conceptual framework for researchers not only to understand the complex biology of cancer but also to make cancer research more potent [3].

Anticancer medications generate by far the most revenue of all therapeutics. The global oncology drug market is expected to reach almost $142 billion in revenue by 2024 [4]. With the challenges of an aging population, the increasing prevalence of chronic illness increased healthcare costs, and the onset of more personalized medicine, drug development must evolve to keep up with the patient’s needs. For a new drug entity, in vitro and in vivo tests must be performed for the determination of toxicity profiles and therapeutic index, which is then followed by the determination of a safe starting dose for phase one trials. In contrast, drug repositioning offers an effective, safe and fast option to control the disease through bypassing several time-consuming safety and dose-related studies. Accordingly, drug repositioning is beneficial for rapidly growing but poorly treated health crises.

**Figure 1:**
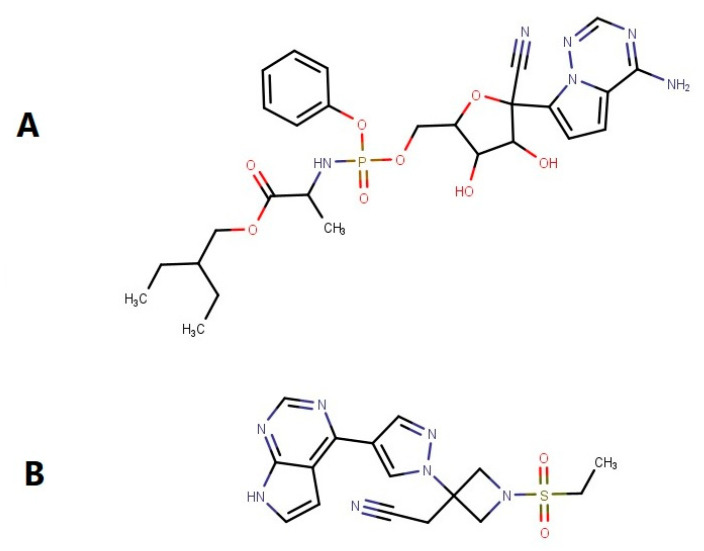
Chemical structure of (**A**) Remdesivir and (**B**) Baricitinib. MarvinSketch was used for drawing, displaying and characterizing chemical structures, MarvinSketch 21.18.0, ChemAxon (https://www.chemaxon.com (accessed on 10 October 2022)).

Remdesivir (RMV, Figure 1A) is a paradigmatic case of effective repositioning. It is a novel nucleotide analog that showed in vitro activity against severe acute respiratory syndrome coronavirus 2 (SARS-CoV-2), and it is the first and only antiviral drug that has obtained the US Food and Drug Administration’s full approval for treating hospitalized COVID-19 patients [5,6]. A number of studies show that the SARS-CoV-2 infection can exacerbate existing diseases, including infectious diseases and cancers. SARS-CoV-2 encoded proteins could cause Kaposi’ssarcoma-associated herpesvirus (KSHV) and Epstein–Barr virus (EBV), aiding in the spread of the virus and the start of oncogenesis [7]. In addition, RMV has demonstrated an anticarcinogenic effect on SKOV3 cells via up-regulation of reactive oxygen species, which suggests that RMV could be a promising reagent for treatment of ovarian cancer [8]. Currently, RMV is only available in injectable form due to complete first-pass elimination of the drug when administered orally through traditional means [9]. Successful bypass of RMV first-pass metabolism upon oral administration is a challenging task but offers valuable benefits for patients. Oral formulation of RMV could ease the capacity constraint of the injectable formulation, offer an affordable, more convenient, easy-to-administer, and potentially effective treatment option for patients.

Baricitinib (BRB), another example of drug repositioning, is a Janus kinase (JAK1/JAK2) inhibitor used for the treatment of rheumatoid arthritis. In addition to immunomodulatory effects, it is thought to have potential antiviral effects through interference with viral entry. In the United States, emergency use authorization has been issued for BRB in combination with RMV in patients with COVID-19 who require oxygen or ventilatory support. In addition, BRB may stop the growth of cancer cells by blocking some of the enzymes needed for cell growth and prevent or lessen the effects of graft-versus-host disease in patients with hematological malignancies [10].

Apart from drug repositioning, the need for natural ingredients in formulation development is rising due to the increased risk of side effects posed by synthetic compounds. Currently, bioactive natural oils play vital roles in developing new drugs, particularly for antitumor, psychoactive, and antimicrobial agents. The exceptional pharmacological benefits of these oils attract scientists to carefully investigate them to obtain novel therapies for many challenging diseases [11]. In this context, black seed is very well known and is considered one of the most promising herbal healing medicines in several Islamic and Arabic countries. Black seed contains over 100 phytochemical constituents which provides synergetic effect strengthening the body’s constitution and supporting the immune system. A late review reported that black seed (along with its oil) can successfully treat more than 129 different human diseases [12,13]. In particular, black seed is reported to be effective against cancer in blood and several body organs. The molecular mechanisms behind its anticancer role is still not clearly understood, however, some studies showed that thymoquinone, one of the major bioactive component in the oil, has antioxidant role, improves body’s defense system, induces apoptosis, and controls the Akt pathway [13,14]. In addition, black seed oil (BSO) contains antioxidants that protect the body from generating free radicals [12]. According to the potential anticancer activity of BSO, it is worthy to investigate its synergistic activity in combination with clinically approved/investigated anticancer drugs.

Self-nanoemulsifying/microemulsifying drug delivery systems (SNEDDS/SMEDDS) can enhance in vivo solubility and drug absorption from the gastrointestinal tract, bypass liver metabolism via lymphatic absorption, and inhibit efflux transport [15]. Bio-SNEDDS is a newer version of SNEDDS developed using biologically active excipients by our group recently. All these phenomena could ultimately improve oral bioavailability and dosage associated adverse effects. Anticancer drug delivery using the SNEDDS has shown promising results for cell viability response with low toxicity. A handful number of research have produced evidence of the successful loading of anticancer agents such as docetaxel, paclitaxel, etoposide, 5 Fluorouracil, and doxorubicin in SNEDDS-based formulations [16,17]. However, excessive surfactant–cosurfactant concentration, lacking predictive in vitro models, and adequate *IVIVC* along with unavailable toxicity data are certain challenges for future researchers [18]. To avail of the benefits of anticancer agents, bio-SNEDDS could contribute to overcoming the challenges and further progression to clinical studies.

In light of the potential data on repositioning of RMV and BRB combination for cancer treatment, we propose the use of bio-SNEDDS to combine both RMV and BRB along with natural bioactive oils (i.e., BSO and FSO). This is an unmet need as there is currently no marketed product of RMV and BRB in combined oral dosage form—only the individual single dosage forms of each drug, which exhibit poor water solubility and low absorption after oral administration and require significant delivery improvement. Within the current study, the optimized bio-SNEDDS formulations were characterized in terms of visual assessment, viscosity, droplet size, and TEM analyses. In addition, the proposed drugs were investigated in terms of their in vitro activity against breast and lung cancer cell lines.

## 2. Results

### 2.1. GC-MS Analysis to Determine Active Phytocomponents of FSO and BSO

**Figure 2:**
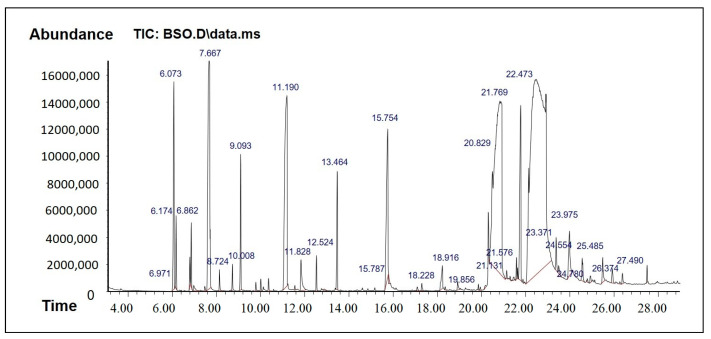
GCMS Chromatogram of BSO.

**Figure 3:**
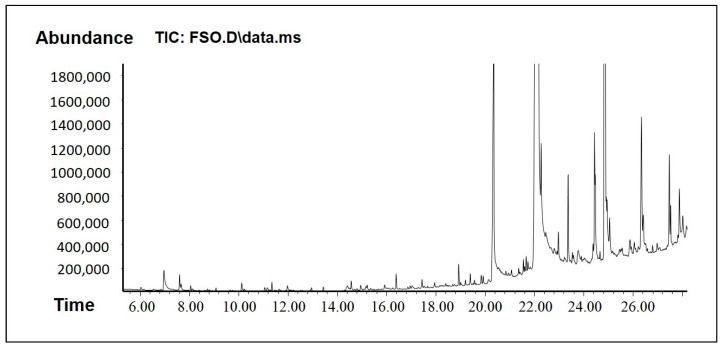
GCMS chromatogram of FSO.

The results from the GC-MS analysis configured unknown compounds which were identified by comparing the spectra with that of the NIST 2008 (Table 1 and Table 2, Figure 2 and Figure 3). The total time required for analyzing a single sample was 29 min. The bioflavonoids and/or bioactive constituents were shown in Table 1 and Table 2. The data from BSO analysis in Table 1 showed a good amount of thymoquinone, which was eluted at 11.19 min. In addition, BSO comprised isoborneol, which has antiviral and antioxidant properties and is a potent inhibitor of herpes simplex virus [19]. BSO was also rich in paeonol, which is a pharmacologically active constituent that possesses anti-inflammatory, antibacterial, and analgesic activities.

Hexadecanoic acid (known as palmitic acid) is a straight-chain, sixteen-carbon, saturated long-chain fatty acid. It has a role as an EC 1.1.1.189 (prostaglandin-E2 9-reductase) inhibitor, a plant metabolite, a Daphnia magna metabolite, and an algal metabolite [20]. Octadec-9-enoic acid (known as oleic acid) belongs to the class of organic compounds known as long-chain fatty acids. Octadec-9-enoic acid has been detected in multiple biofluids, such as blood and urine. Within the cell, octadec-9-enoic acid is primarily located in the cytoplasm. It is an unsaturated fatty acid that is the most widely distributed and abundant fatty acid in nature [21].

FSO, on the other hand, showed some bioactive constituents, namely p-cymenene, phytol, and squalene (Table 2). These bioactive constituents are reported to act as analgesic, antinociceptive, immunomodulatory, and neuroprotective agent. They have the pharmacological properties including antioxidant, anti-inflammatory, antiviral, antitumor, antibacterial, and antifungal activities. The anticancer effects of p-cymene are related to mechanisms such as the inhibition of apoptosis and cell cycle arrest. In addition, FSO has many other fatty acids and polar compounds which are active biologically and also support in enhancing drug solubility.

### 2.2. Visual Assessment of the Formulations

**Figure 4:**
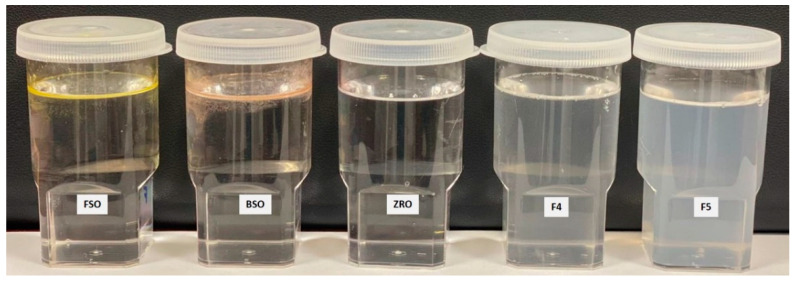
Physical appearance of the formulation upon aqueous dispersion (anhydrous formulation was diluted at a 1:1000 dilution factor).

The resulting emulsions of F1–F3 formulations experienced poor homogeneity (oil floats on the water). In particular, F1 (FSO) and F2 (BSO) showed yellowish and reddish colors on the water surface (Figure 4), respectively. For poor dispersibility (large droplets) and hazy appearance, these F1–F3 formulations were not categorized as bio-SNEDDS and were instead named as oil-only formulations. However, due to the presence of nonionic surfactant Tween 80 (refined) and polar mixed mono- and di-glycerides (Imwitor 988) in formulations F4 and F5, the emulsions produced were fine (semi-transparent systems with no discernible particulates) following aqueous dispersion. These two formulations produced homogeneous emulsion droplets that were slightly bluish in appearance. However, the anhydrous state of F4 showed partial physical separation over time and was not completely miscible, which might be due to the presence of the natural crude oil FSO. Accordingly, only F5 showed complete excipients miscibility (in an anhydrous state) and homogenous aqueous dispersion (upon dilution) and, therefore, were tagged as bio-SNEDDS.

### 2.3. Robustness to Dilution

After dilution with different aqueous media, the resultant aqueous dispersion showed more transparency for higher-dilution (1:1000) nanoemulsions, which generally remained stable after 24 h. On the other hand, the lower dilutions—1:10 and 1:100—showed a turbid appearance and underwent phase separation upon storage that tended to homogenize again upon gentle mixing (Table 3).

### 2.4. Thermodynamic Stability

As summarized in Table 4, all of the tested formulations showed reversible phase separation after centrifugation, heat–cool cycles, and freeze–thaw cycles and homogenize again after gentle mixing.

### 2.5. Droplet Size, Zeta Potential Measurement of the Bio-SNEDDS

**Figure 5:**
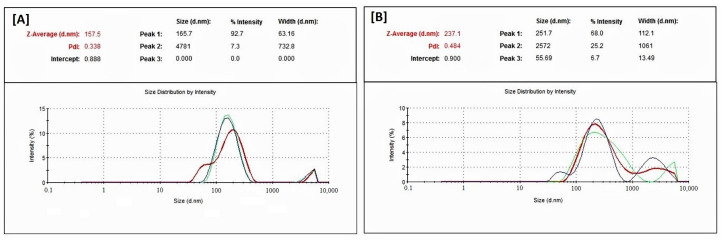
Malvern zetasizer particle size distribution data for F4 systems and F5 bio-SNEDDS; (**A**) FSO/I988/T80R (35/15/50) %*w*/*w*/*w* and (**B**) BSO/I988/T80R (35/15/50) %*w*/*w*/*w*, respectively.

The droplet size data (Table 5 and Figure 5) showed that BRB and RMV bio-SNEDDS prepared using FSO (F4-) and BSO (F5-Bio-SNEDDS) exhibited low average particle sizes of 151.60 ± 1.51 and 247.03 ± 9.18 nm, respectively. In addition, F4 and F5 showed polydispersity indices (PDI) of 0.329 ± 0.059 and 0.441 ± 0.038, respectively, which reflects wide size distribution and high variability. On the other hand, the zeta potential values of F4-systems and F5-Bio-SNEDDS were negatively and positively charged, respectively. F5-bio-SNEDDS showed a higher zeta potential (absolute) value compared to F4-systems. The pure oils such as FSO, BSO and ZRO were not well dispersed in aqueous media and thus formed very crude droplets (>5 µm) with high PDI values (≥1).

### 2.6. Equilibrium Solubility and Drug Loading in the Bio-SNEDDS

Poor solubility and their consequent absorption prevents any drug from entering into clinical trials. The solubilities of RMV and BRB in anhydrous bio-SNEDDS formulations were shown in Table 6. The bio-SNEDDS were able to solubilize the maximum amount of drug and promoted high self-emulsification efficiency compared to oil only formulations (F1–F3).

The anhydrous F4 systems and F5 bio-SNEDDS formulations showed 6.21 mg/g and 7.04 mg/mL BRB solubility, respectively, whereas RMV solubility was slightly increased in both formulations as 8.20 mg/g and 10.93 mg/mL, respectively (Table 6).

Accordingly, the pure-drug BRB and RMV were loaded (5 mg/g) in both F4 systems and F5 bio-SNEDDS formulations, which was below the equilibrium solubility of the formulations. These concentrated bio-SNEDDS were used in all the further experimental investigations.

### 2.7. Viscosity of the Bio-SNEDDS Formulations

The viscosities of self-nanoemulsifying formulation systems were monitored by standard rheological techniques. The optimized formulations containing bioactive oil and surfactant represented by F4 systems and F5 bio-SNEDDS has minimum viscosity of 0.717 Cp and 0.693 Cp for drug-free bio-SNEDDS, respectively (Table 7). However, the drug-loaded F4 systems and F5 bio-SNEDDS (RMV and BRB) formulations yielded slightly higher viscosities of 0.777 Cp and 0.717 Cp, respectively.

The overall viscosity data of anhydrous formulation systems and bio-SNEDDS confirmed the requirements of SNEDDS characteristics. The viscosity of formulations is dependent on the use of lipid oils and surfactants in the delivery systems. In the current investigation, the bio-SNEDDS were less viscous and immediately released a formulation with very fast dispersion properties.

### 2.8. TEM Analysis

**Figure 6:**
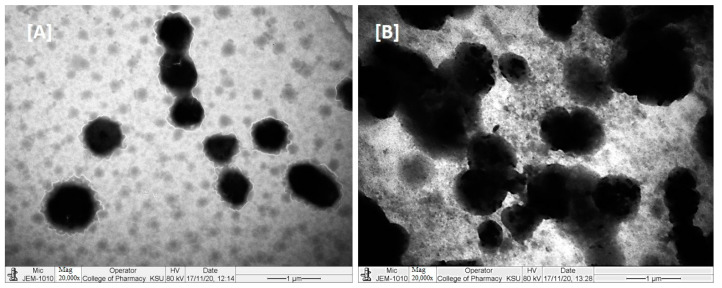
TEM images of RMV- and BRB-loaded representative formulations ((**A**) represents F4-formulation systems and (**B**) represents F5-bio-SNEDDS).

TEM micrographs of RMV and BRB loaded bio-SNEDDS formulations are shown in Figure 6. The TEM suggested a valuable interpretation of the surface morphology and globule size of the bio-SNEDDS. In the image, it was apparent from (A) that globules of the representative F4-systems and F5-bio-SNEDDS were well dispersed and had spherical shape. However, the F5-bio-SNEDDS was found to be larger and irregular in size.

### 2.9. Effect of Different Concentrations of Pure Oils and Their Different Formulations on the Proliferation of Breast Cancer Cell Lines

**Figure 7:**
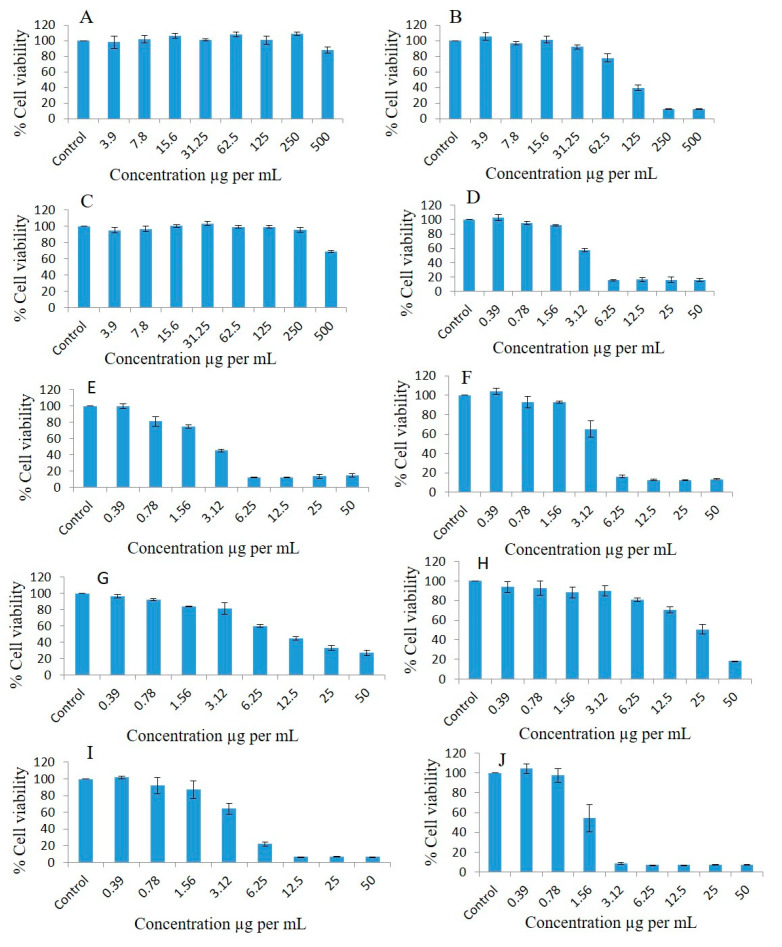
Effects of different concentrations of pure FSO (**A**), BSO (**B**), ZRO (**C**), F4- systems-loaded RMV (**D**), F4-systems-loaded BRB (**E**), F4-systems blank (drug free systems) (**F**), pure BRB (**G**), pure RMV (**H**), F4-systems-loaded with RMV and BRB (**I**), F5-bio-SNEDDS loaded with RMV and BRB (**J**) on the cell viability percentage of the MDA-MB-231 breast cancer cell line as measured using MTT 72 h following exposure.

Breast cancer cell lines such as MDA-MB-231 were used to observe the antiproliferative effects at different concentrations of pure oils and various formulations systems and Bio-SNEDDS by conducting a dose escalation study utilizing MTT-based viability assays. FSO (alone) could not inhibit the proliferation of breast cancer cell line as illustrated in Figure 7A. Interestingly, MDA-MB-231 treated with different concentrations of BSO (alone) showed concentration-dependent % cell viability of 105.34, 96.8, 101.36, 91.86, 77.82, 39.65, 12.45, and 12.42 at doses of 3.9, 7.8, 15.6, 31.25, 62.5, 125, 250, and 500 µg/mL, respectively, compared to the controls after 72 h treatment (Figure 7B). ZRO (alone) could not inhibit the proliferation of the breast cancer cell line (Figure 7C). As illustrated in Figure 7D, the F4 formulation systems loaded with RMV-inhibited MDA-MB-231 cell line had % cell survival 102.61, 95.00, 91.98, 57.3, 15.41, 16.34, 16.3, and 15.75 at doses of 0.39, 0.78, 1.56, 3.12, 6.25, 12.5, 25, and 50 µg/mL, respectively compared to the controls.

The F4 systems loaded with BRB inhibited MDA-MB-231 cell line proliferation 0.99-, 0.81-, 0.74-, 0.45-, 0.12-, 0.12-, 0.13- and 0.14-fold, and cell viability percentages were 99.79, 81.16, 74.84, 45.34, 12.34, 12.14, 13.67, and 14.68, respectively, at doses of 0.39, 0.78, 1.56, 3.12, 6.25, 12.5, 25, and 50 µg/mL, respectively (Figure 7E). The F4 Blank formulation (drug free systems) showed the cell viability percentages of the MDA-MB-231 cell line as 92.95, 92.82, 65.2, 16.3, 12.82, 12.74, and 13.54 at doses of 0.39, 0.78, 1.56, 3.12, 6.25, 12.5, 25, and 50 µg/mL, respectively, compared to the controls (Figure 7F).

The cell viability percentages of pure BRB in the MDA-MB-231 cell line were 96.4, 91.95, 83.99, 81.34, 59.85, 44.75, 32.91, and 27.03 at doses of 0.39, 0.78, 1.56, 3.12, 6.25, 12.5, 25, and 50 µg/mL, respectively, compared to the controls (Figure 7G). The pure-RMV-inhibited MDA-MB-231 cell line’s cell viability percentages were 93.99, 92.64, 88.2, 89.97, 80.88, 70.65, 50.51, and 18.08 at doses of 0.39, 0.78, 1.56, 3.12, 6.25, 12.5, 25, and 50 µg/mL, respectively, compared to the controls (Figure 7H).

The F4-systems loaded with combined RMV and BRB inhibited MDA-MB-231 cell line proliferation 1.02-, 0.99-, 0.93-, 0.69-, 0.22-, 0.06-, 0.06-, and 0.06-fold, and the cell viability percentages were 102.03, 92.29, 87.18, 64.41, 22.14, 6.79, 6.81, and 6.65, respectively, at doses of 0.39, 0.78, 1.56, 3.12, 6.25, 12.5, 25, and 50 µg/mL, respectively, compared to the controls (Figure 7I). The F5-SNEDDS loaded with combined RMV and BRB inhibited MDA-MB-231 cell line proliferation 1.05-, 0.98-, 0.66-, 0.08-, 0.07-, 0.07-, 0.07-, and 0.07-fold, and the cell viability percentages were 104.23, 97.39, 54.27, 8.55, 7.22, 7.16, 7.24, and 7.21, respectively, at doses of 0.39, 0.78, 1.56, 3.12, 6.25, 12.5, 25, and 50 µg/mL, respectively, compared to the controls (Figure 7J).

The concentrations causing 50% inhibition of growth of breast cancer cells (IC_50_) were calculated using a trendline equation and are presented in Table 8.

### 2.10. Effect of Different Concentrations of Pure Oils and Their Different Formulations on the Proliferation of A549 Lung Cancer Cell Lines

**Figure 8:**
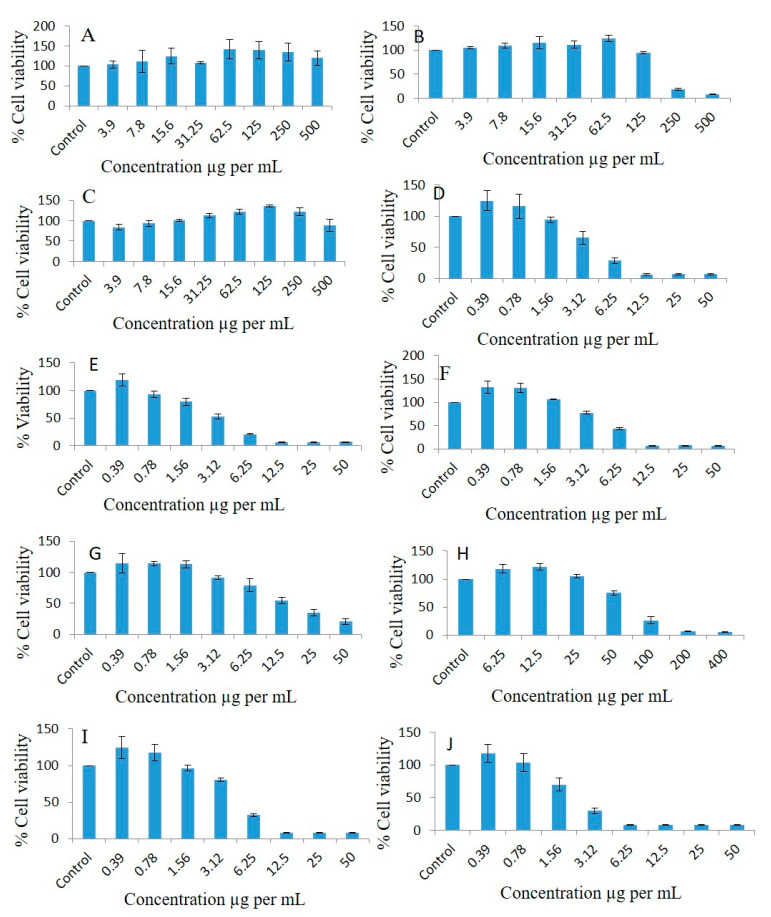
Effects of different concentrations of FSO (**A**), BSO (**B**), ZRO (**C**), F4-systemsloaded RMV (**D**), F4-systems-loaded BRB (**E**), F4-blank (drug free systems) (**F**), pure BRB (**G**), pure RMV (**H**), F4-systems-loaded RMV and BRB (**I**), F5-bio-SNEDDS-loaded RMV and BRB (**J**) on the cell viability percentages of the A549 lung cancer cell line as measured using MTT 72 h following exposure.

FSO could not inhibit the proliferation of the A549 lung cancer cell line (Figure 8A). Interestingly, BSO inhibited proliferation of A549, and the cell viability percentages were 104.81, 109.65, 114.55, 111.11, 124.04, 94.49, 18.86, and 7.58 at doses of 3.9, 7.8, 15.6, 31.25, 62.5, 125, 250, and 500 µg/mL, respectively, compared to the controls (Figure 8B). ZRO could not inhibit the proliferation of the lung cancer cell line (Figure 8C). As illustrated in Figure 8D, the F4-systems-loaded RMV inhibited lung cancer cell line proliferation, and the cell viability percentages were 124.73, 116.37, 94.23, 65.59, 28.42, 6.37, 6.49, and 6.56 at doses of 0.39, 0.78, 1.56, 3.12, 6.25, 12.5, 25, and 50 µg/mL, respectively, compared to the controls. The F4-systems-loaded BRB inhibited lung cancer cell line proliferation, and the cell viability percentages were 118.28, 92.67, 79.39, 52.98, 20.69, 6.18, 6.21, and 6.84 at doses of 0.39, 0.78, 1.56, 3.12, 6.25, 12.5, 25, and 50 µg/mL, respectively, compared to the controls (Figure 8E).

The F4-blank (drug free systems) inhibited the lung cancer cell line, and the cell viability percentages were 132.37, 130.65, 106.01, 77.54, 43.12, 6.8, 7.2, and 7.09 at doses of 0.39, 0.78, 1.56, 3.12, 6.25, 12.5, 25, and 50 µg/mL, respectively, compared to the controls (Figure 8F). The cell viability percentages of pure BRB drug powder in the lung cancer cell line were 114.28, 114.24, 113.03, 91.45, 78.97, 54.36, 35.03, and 20.63 at doses of 0.39, 0.78, 1.56, 3.12, 6.25, 12.5, 25, and 50 µg/mL, respectively, compared to the controls (Figure 8G). Similarly, the cell viability percentages of pure RMV drug powder in the lung cancer cell line were 118.23, 121.48, 104.6, 75.38, 26.33, 6.77, and 5.67 at doses of 6.25, 12.5, 25, 50, 100, 200, and 400 µg/mL, respectively, compared to the controls (Figure 8H). The F4-systems-loaded RMV and BRB inhibited lung cancer cell line proliferation 1.23-, 1.17-, 0.96-, 0.8-, 0.32-, 08-, 0.07, and 0.08-fold, and the cell viability percentages were 124.2, 117.92, 96.31, 80.44, 32.24, 8.09, 7.9, and 8.35, respectively, at doses of 0.39, 0.78, 1.56, 3.12, 6.25, 12.5, 25, and 50 µg/mL, respectively, compared to the controls (Figure 8I). The F5-bio-SNEDDS-loaded RMV and BRB inhibited lung cancer cell line proliferation 1.16-, 1.02-, 0.69-, 0.29-, 0.08-, 07-, 0.08-, and 0.08-fold, and the cell viability percentages were 117.18, 103.26, 69.96, 29.56, 8.3, 7.97, 8.3, and 8.22, respectively, at doses of 0.39, 0.78, 1.56, 3.12, 6.25, 12.5, 25, and 50 µg/mL, respectively, compared to the controls (Figure 8J).

### 2.11. Effect of Different Concentrations of Pure Oils and Their Different Formulations on the Proliferation of Normal Human Fibroblasts Cell Lines

**Figure 9:**
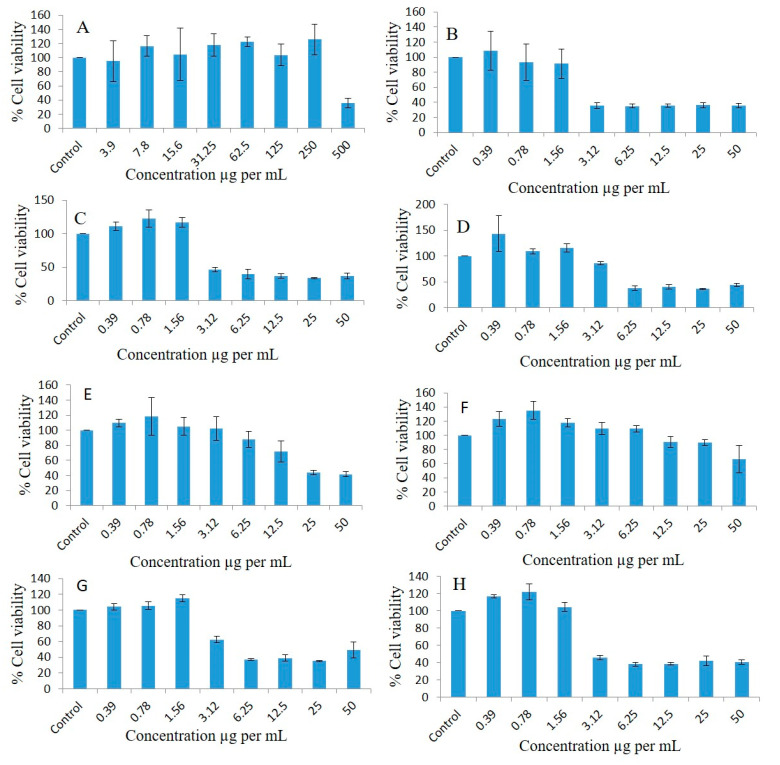
Effects of different concentrations of BSO (**A**), F4-systems-loaded RMV (**B**), F4-systems-loaded BRB (**C**), F4-blank (drug free systems) (**D**), pure BRB (**E**), pure RMV (**F**), F4-systems-loaded RMV and BRB (**G**), and F5-bio-SNEDDS-loaded RMV and BRB (**H**) on the cell viability percentages of the human fibroblast cell line as measured using MTT 72 h following exposure.

As FSO and ZRO were unable to inhibit the proliferation of the MDA-MB-231 breast cancer and A549 lung cancer cell lines (Figure 7 and Figure 8), these oils were excluded in the human fibroblasts cell line studies. As illustrated in Figure 9A, BSO inhibited proliferation, and the cell viability percentages were 95.07, 116.29, 104.69, 117.84, 122.47, 103.72, 125.53, and 35.62 at doses of 3.9, 7.8, 15.6, 31.25, 62.5, 125, 250, and 500 µg/mL, respectively, compared to the control. The F4-systems-loaded RMV (Figure 9B) showed the cell viability percentages as 108.57, 93.2, 91.2, 35.49, 35.05, 35.68, 36.37, and 35.58 at doses of 0.39, 0.78, 1.56, 3.12, 6.25, 12.5, 25, and 50 µg/mL, respectively compared to the control. Similarly, F4-formulation-systems-loaded BRB also showed the cell viability percentages as 111.21, 122.39, 117.12, 46.56, 39.66, 36.72, 33.8, and 36.76 at doses of 0.39, 0.78, 1.56, 3.12, 6.25, 12.5, 25, and 50 µg/mL, respectively, compared to the control (Figure 9C).

As illustrated in Figure 9D, the cell viability percentages of F4-blank (drug-free systems) were 143.48, 109.04, 115.52, 85.91, 37.51, 40.16, 36.25, and 44.15 at doses of 0.39, 0.78, 1.56, 3.12, 6.25, 12.5, 25, and 50 µg/mL, respectively, compared to the control. The pure BRB drug powder (Figure 9E) showed cell viability percentages of 109.79, 118.13, 105.07, 101.93, 87.89, 71.73, 43.77, and 41.58, whereas the pure RMV drug powder showed slightly higher cell survival percentages of 123.43, 135.13, 117.85, 109.67, 109.49, 90.4, 89.56, and 66.37 (Figure 9F) at doses of 0.39, 0.78, 1.56, 3.12, 6.25, 12.5, 25, and 50 µg/mL, respectively, compared to the controls.There was not any significant difference in cell survival noticed between F4-systems- and F5-bio-SNEDDS-loaded RMV and BRB. As illustrated in Figure 9G,H, F4-systems-loaded RMV and BRB provided cell viability percentages of 104.14, 105.42, 114.84, 62.56, 36.89, 38.83, 35.12, 49.23, and F5-bio-SNEDDS-loaded RMV and BRB provided 116.73, 122.18, 104.5, 45.6, 38.09, 38.49, 41.99, and 40.7 at doses of 0.39, 0.78, 1.56, 3.12, 6.25, 12.5, 25, and 50 µg/mL, respectively, compared to the controls.

IC_50_ value of MDA-MB-231 breast cancer, A549 lung cancer, and human fibroblast cell lines were calculated using a trendline equation and are presented in Table 8. Among the screened bioactive oils, BSO was the only oil to show a concentration-dependent growth inhibition of both breast and lung cancer cell lines. Pure BRB is more active compared to pure Remdesivir, as confirmed by the significantly lower (*p* < 0.05) IC50 of BRB in both breast and lung cancer cells. Interestingly, drug-free and drug-loaded F4-formulation systems (either loaded with one or both drugs) along with combined drug-loaded F5-bio-SNEDDS showed similar antiproliferative activity of breast cancer cells, as confirmed by the nonsignificant difference (*p* > 0.05) between IC-50 of all these formulations. Similar findings were observed in lung cancer cells, with minor differences. It is worth mentioning that the formulation systems and bio-SNEDDS (drug-free or drug-loaded) showed superior antiproliferative activity compared to pure RMV and pure BRB.

## 3. Materials and Methods

### 3.1. Materials

Remdesivir (RMV) reference standard (purity 99.9%) was purchased from Zhejiang Hongyuan Pharmaceutical Co. Ltd. (Zhejiang, China). Baricitinib (BRB) reference standard (purity > 99.8%) was purchased from Biocompounds Pharmaceutical Inc. (Songjiang, shanghai, China). Imwitor 988 (I988, medium chain mono and diglycerides) and nonionic surfactant Tween 80 Refined (T80R) were obtained from BASF (Ludwigshafen, Germany) and Croda Inc. (Princeton, NJ, USA), respectively. The Milli-Q highly purified water was used from a Milli-Q Integral Water Purification System (Millipore, Bedford, MA, USA). All other chemicals and reagents used in the studies were analytically pure.

MDA-MB-231 (breast cancer cells), A549 (lung cancer cells), and human fibroblast cell lines were used in this study, were obtained from the American Type Culture Collection (ATCC, Manassas, VA, USA). All tissue culture media and materials—including DMEM, L-glutamine, and penicillin/streptomycin—and Fetal Bovine Serum were obtained from Gibco Inc. (Brooklyn, NY, USA). Cell culture flasks (25 and 75 cm^2^) with vent cap, Falcon^®^ 15 and 50 mL polystyrene centrifuge tubes and sterile individually wrapped StripetteTM serological polystyrene pipettes were purchased from Corning^®^ USA. All protein chemistry reagents and buffers were obtained from Bio-Rad Laboratories GmbH (Munich, Germany).

### 3.2. Plant Material and Extraction of Bioactive Oils

The methods of collection, extraction, and standardization of black seed oil (BSO) and zanthoxylum rhetsa seed oil (ZRO) were explained in detail in our previous publication [11,22]. BSO was obtained from the seeds of *Nigella sativa* (*N. sativa*) Linn. (black seeds), family Ranunculaceae and ZRO were obtained from dried fruits of the Roxb DC plant, which belongs to the family Rutaceae, via the cold press/stem distillation method. Fenugreek seed oil (FSO) and BSO were collected from Kaligonj, Gazipur, Bangladesh and cold pressed. The extracted oil was filtered and stored in a screw-capped amber glass bottle for further use in bio-SNEDDS development.

### 3.3. GC-MS Analysis to Determine Active Phytocomponents of FSO and BSO

#### 3.3.1. GC-MS Analysis Method

GC-MS analysis of natural compounds BSO and FSO was carried out as described: The components of the oils were identified by matching the peaks with the National Institute of Standard and Technology Library (NIST) Mass Spectral Library.

#### 3.3.2. GC-MS Instrumentation

Agilent GC 7890A combined with a triple-axis detector 5975 C single quadrupole mass spectrometer was used for GC-MS analysis. The chromatographic column was an Agilent HP 5MS column (30 m × 0.25 mm × 0.25 µm film thickness), with high-purity helium as the gas carrier, at a flow rate of 1 mL/min. The injector temperature was 280 °C, and it was equipped with a splitless injector at 20:1. The source temperature of MS was set at 230 °C, and the quad temperature was at 150 °C. The oven temperature was initially at 40 °C (held for 1 min) and was increased to 150 °C at 10° C min^−1^ (held for 1 min), then increased further to 300 °C at 10 °C min^−1^ for 1 min. The scan range was set at 40 to 600 mass ranges at 70 eV electron energy and the solvent delay was set to 3 min.

### 3.4. Selection of Excipients and Bio-SNEDDS Development

Selection of the correct excipient is the key to successful bio-SNEDDS formulation development, which can influence the droplet size and absorption of these systems. The excipients used to develop bio-SNEDDS in this research were natural oils, hydrophilic surfactants, and water-soluble cosolvents. Different ratios of oils and surfactants were blended to design bio-SNEDDS formulations (Table 9) [23]. Lipid formulation classification systems (LFCS) was used as a framework to develop the bio-SNEDDS without performing any trial and error. LFCS was established by Pouton in terms of the performance of formulation dispersion and digestion. According to LFCS, four “Type”s of lipid-based formulations categorized, from Type I-oil rich (water insoluble) to Type IV-surfactant rich (water soluble) formulation systems. LFCS Class III-IV systems usually produce bio-SNEDDS formulation. In this study, both bio-systems were formulated using four components—the bioactive oils BSO, FSO, mixed glycerides I988 and surfactant T8OR—and by changing their combinations.

### 3.5. Euilibrium Solubility and Drug Loading into Bio-SNEDDS

The loading of BRB and RMV in five lipid-based formulations was determined using the simple “shake flask” drug solubility method. Samples for the equilibrium solubility experiment were prepared by adding excess amount of BRB and RMV in the formulations at room temperature (23 °C ± 1 °C). After 7 days of keeping the samples at 37 °C in a dry heat incubator, they were removed for centrifugation (at 13,000 rpm for 10 min) to separate excess solid drug from solubilized drug. Then, an aliquot of approximately 50 mg was weighed and diluted in 50 mL of acetonitrile. The solubilized amount of BRB and RMV was analyzed using a validated ultra-high-performance liquid chromatography (UHPLC) method.

The dosage forms of the F4 systems and F5 bio-SNEDDS were prepared by loading BRB and RMV in combination. BRB (5 mg) and RMV (5 mg) were dissolved in formulation by vortexing for 5 min and then sonication for an extra 5 min at 37 °C. Mixtures were centrifuged at 13,200× *g* for 5 min to ensure that drugs were solubilized completely. The drug-loaded formulations were stored in screw-capped glass vials and used for further studies.

### 3.6. Visual Assessment of the Formulations

To examine the formulations’ miscibility, homogeneity, and appearance, a visual assessment method was taken as a guide [23,24]. The physical stability of the emulsion produced by aqueous dilution was determined by visual examination of all representative formulations.

### 3.7. Robustness to Dilution

The anhydrous formulations were diluted with distilled water, 0.1 N HCl, or phosphate buffer (pH 6.8) ≈ 10, 100, and 1000 times. The mixture was vortexed for at least 30 s and stirred for at least 2 min at 1000 rpm after each dilution. To check for any phase separation or drug precipitation, each diluted sample was kept at room temperature for 24 h [25].

### 3.8. Thermodynamic Stability

Studies of thermodynamic stability were carried out to determine how temperature and centrifugation affected the stability of SNEDDS. To evaluate physical stability, each formulation was diluted with water (1:100) and centrifuged at 5000 rpm for 30 min. Each formulation also underwent four alternative heat–cool cycles between 4 °C and 40 °C with storage at each temperature for at least 48 h. Additionally, the formulations were subjected to four alternative freeze–thawing cycles at −20 °C and 22 ± 2 °C after being stored at each temperature for a minimum of 48 h. At the conclusion of each cycle, the physical appearance and phase separation were evaluated [25].

### 3.9. Measurement of Droplet Size, Polydispersity Index (PDI), and Zeta Potential

The droplet size, PDI, and zeta potential analyses were performed for bio-SNEDDS according to the previous studies [22,24,26,27]. For sample preparation, the anhydrous formulations were diluted with milliQ water at a ratio of 1:1000 (*w*/*w*) and mixed for 1 min before test. The mean droplet size, distribution, and PDI of the samples were measured using a Zetasizer Nano ZS analyzer (Model ZEN3600, Malvern Panalytical Ltd., Malvern, UK). The zeta potential of each bio-SNEDDS was evaluated using laser Doppler velocimetry (LDV) mode at 25 °C.

### 3.10. Viscosity of the Bio-SNEDDS Formulations

The viscosity of bio-SNEDDS systems was monitored using standard rheological techniques. The anhydrous formulation was diluted with Milli-Q water at a 1:1000 dilution factor. The diluted sample solution was filled in a 50 mL chamber and then measured using a small sample adapter of Brookfield cone and plate rheometer (Model LV2, Brookfield Engineering Laboratories, Stoughton, MA, USA) at room temperature (25 °C). The sample was repeated in triplicate.

### 3.11. Transmission Electron Microscopy (TEM)

Bright field transmission electronic images of the representative SNEDDSs were taken using JEOL, JEM1010 (JEOL Ltd., Akishima, Tokyo, Japan). Samples for TEM analysis were prepared via 1 in 10 dilution with water. The instrument was operated at 300 keV, and the solution of the sample SNEDDS were measured on copper-grid-supported Formvar films.

### 3.12. Cell Lines and Culturing Conditions

MDA-MB-231 (breast cancer), A549 (lung cancer), and normal human fibroblasts cell lines were cultured in full medium containing DMEM supplemented with 10% FBS, 1% antimycotic at 37 °C, and 5% CO_2_ in 75 cm^2^ tissue culture flasks. For the cytotoxicity assessment, cells were trypsinized (Trypsin 0.05%/0.53 mM EDTA) and reaction was stopped by adding full medium to collect all cells. Then, cells were seeded (2 × 10^3^ cell/well in 200 µL of medium) into 96-well plates (Gibco, NY, USA) for 24 h before being treated with different concentrations of pure oils, drugs, and/or their different formulations for another 72 h [28].

### 3.13. Anti-Proliferative Activity via MTT Assay

The cytotoxic effect of different concentrations of pure oils, pure drugs, and bio-SNEDDS formulations were evaluated by testing the capacity of the reducing enzymes present in viable cells to convert 3-[4,5-dimethylthiazol-2-yl]-2,5-diphenyltetrazolium bromide (MTT) to formazan crystals. After 72 h of incubation with different concentrations of pure oil, such as 3.9, 7.8, 15.6, 31.25, 62.5, 125, 250, 500, and 1000 µg/mL beside incubation with different concentrations of their formulations, such as 0.39, 0.78, 1.56, 3.12, 6.25, 12.5, 25, and 50 µg/mL, the media were discarded and adherent cells were incubated with 100 µL/well MTT at a concentration of 0.5 mg/mL prepared in PBS and subsequently incubated at 37 °C for additional 3 h at 37 °C under dark conditions [28]. Then, 100 µL isopropyl alcohol was added per well to dissolve the purple formazan crystals with the help of shaking for another 2 h at room temperature. Subsequently, the absorbance was measured at 549 nm using an ELX 800 BioTek microplate reader (BioTek Instruments, Winooski, VT, USA). The results were analyzed in triplicate, and the viability percentage was calculated. The cytotoxicity of different formulations was determined by testing the capacity of the reducing enzymes present in the viable cells to convert MTT to formazan crystals [29]. Concentrations causing 50% inhibition of growth of MDA-MB-231 breast cancer cells and A549 lung cancer cells (IC50) were calculated by use of a Microsoft Excel trendline equation [28].

### 3.14. Statistical Analysis

One-way analysis of variance (IBM SPSS Statistics 26^®^, ANOVA) followed by post hoc tests (LSD) were applied to compare the IC50 of different formulations. A value of *p* < 0.05 was considered significant throughout the study [30].

## 4. Discussion

This article discusses the methods of preparation, characterization, encapsulation of RMV and BRB; the effect of physicochemical properties of bio-SNEDDS; and their use in cancer therapy.

Interestingly, bio-SNEDDS can potentially enhance the oral delivery of a poorly water-soluble drug through simultaneous portal blood absorption and lymphatic delivery. The relative bioavailabilities of similar lipid-based systems with normal and blocked chylomicron flow were about 210% and 164%, respectively, in comparison to aqueous drug suspension [31]. The significant increases in dissolution and lymphatic delivery propose that these systems could be a promising technique to improve oral RMV/BRB delivery and bypass its first-pass metabolism through lymphatic delivery. In this study, an attempt was made to design an effective oral bio-SNEDDS through the combination of the repositioned drugs (RMV and BRB) and natural bioactive oil (BSO, FSO, and/or ZRO).

Although microemulsions and nanoemulsions are physicochemically distinct systems, their similar terminologies, structural features, and manufacturing methods frequently cause them to be mistakenly identified, sometimes even based solely on their droplet size [32]. It appears from the terms that the nanoemulsions possess smaller droplet sizes than microemulsions, but in reality, the opposite is true. Although the term “micro” suggests that the microemulsion droplets are in the micro range, their actual size is below 100 nm. Similarly, “nano” refers to the size of nanoemulsion droplets in nanometers, which is accurate given that they are typically smaller than 300 nm [33]. The main difference is that nanoemulsions are thermodynamically unstable but kinetically stable dispersions, while microemulsions are thermodynamically stable dispersions [34]. In addition, microemulsions generally tend to produce narrow particle size distributions, while nanoemulsions usually present wide size distributions or even multiple peaks because all the droplets are not of the same size.

In the current study, F5 showed an average droplet size of 247 nm with 0.44 PDI and multiple peaks (Table 5, Figure 5), which reflects a wide size distribution and suggests that the formulation is probably SNEDDS rather than SMEDDS. Interestingly, F4 and F5 showed significant changes in physical appearance upon increasing dilution magnitude (Table 3). These findings might not match a recent study that suggested that nanoemulsions should be robust enough to undergo dilution without rapid change in droplet size [34]. Most importantly, F4 and F5 are thermodynamically unstable, as reported in Table 4, and hence are not categorized as SMEDDS. Based on the overall formulation characteristics, F5 seems to be better at matching the physicochemical properties of SNEDDS.

The visual assessment findings reflect the superior role of non-ionic hydrophilic surfactants and polar mixed mono- and di-glycerides (Imwitor 988) in enhancing the self-nanoemulsification quality. The low droplet sizes of F4 and F5 bio-SNEDDS are desirable and can be attributed to one or more of the following reasons below:

(a) The use of high proportion of hydrophilic excipients in the formulation (Type III system) [35].
(b) The inclusion of bioactive oils, which possess good self-emulsification properties as BSO [11].
(c) The inclusion of the polar mixed glycerides in the formulation.
(d) The inclusion of the highly purified and highly hydrophilic surfactant, refined T80 (HLB = 15), which was suggested to adhere to the drug particles’ surfaces, forming a protective layer that reduces solid–liquid interfacial tension, preventing agglomeration of the particles [36].

The overall TEM images suggest that the droplets were in the nanosize range (200–350 nm), which was also evident from the particle size distribution data (Table 5).

In particular, F5 showed acceptable zeta potential values irrespective of the charge [37]. However, this might not significantly affect SNEDDS formulation performance because they are anhydrous systems that are expected to mix with aqueous media in the body GIT only. Accordingly, long-term emulsion stability is not critical for these systems. The overall viscosity data of anhydrous formulation systems and bio-SNEDDS confirmed the requirements of SNEDDS characteristics. The viscosity of formulations is dependent on the use of lipid oils and surfactants in the delivery systems. In the current investigation, F4 and F5 were less viscous and immediately released formulations with fast dispersing properties.

From the overall solubility data, it was confirmed that two formulations were able to solubilize the higher amounts of BRB and RMV with better aqueous dispersibility compared to oily formulations. The high polarity of the bio-SNEDDS (polar glycerides and water-soluble surfactant) could be the driving force for the solubility improvement of BRB and RMV. Therefore, F4 and F5 were chosen for further characterization and cell line studies.

Furthermore, the current study assessed the antiproliferative activities (cell viability percentage) of different bio-SNEDDS formulations of FSO and BSO against the MDA-MB-231 breast cancer cell line, A549 lung cancer cell line, and human fibroblast cell line. Pure BSO showed concentration-dependent growth inhibition of both breast and lung cancer cell lines. These findings are in agreement with previous studies that reported BSO activity against cancer in several body organs [14]. In addition, the higher IC50 of BSO in normal human fibroblasts cell line reflects its good tolerability in normal fibroblast cells. Surprisingly, F4 systems and F5 bio-SNEDDS formulations (drug-free or drug-loaded) showed superior antiproliferative activity compared to pure RMV and pure BRB. In particular, drug-free F4 formulation systems and F5 bio-SNEDDS showed similar antiproliferative activity to RMV, BRB, and RMV + BRB-loaded bio-SNEDDS, which could be due to one or more of the following:

(1) Drug-free formulation systems and bio-SNEDDS possess superior anti-proliferative activity compared to RMV and BRB.
(2) The very low RMV or BRB concentration in bio-SNEDDS (5 mg/g) could be remarkably masked by bio-SNEDDS activity.
(3) The cosurfactant I988 and/or surfactant refined Tween 80 might possess anti-proliferative activity against breast and lung cancer cells [38].

The superior activity of drug-free F4 systems and F5 bio-SNEDDS highlights the superior benefits of comprising bioactive components particularly when properly formulated into efficient bio-SNEDDS. However, further studies are warranted to deeply interpret the activity of bio-SNEDDS bioactive oils, surfactants, and/or cosurfactants.

The proposed strategy of developing bio-SNEDDS for the combined dosage form of RMV and BRB could enable us to develop a platform technology which could be applied to other drugs for combined therapy during any future outbreak. The combined dose might involve lower drug concentrations compared to single doses, thus posing a lower risk of side effects. Such formulations could be very flexible in producing and enabling the development of a diverse range of dosage forms, such as encapsulated in soft and/or hard gelatin capsules, oral solutions (palatable in taste, suitable for pediatric patients), and solid dosage form (powder, tablets, pellets). Therefore, bio-SNEDDS have remained as extensively demanding, effective alternatives to conventional emulsion or tablets (hard to swallow for children). No sophisticated technology infrastructure is required to use this formulation technique, and the products could be readily developed with readily available pharmaceutical production equipment.

The oral bio-SNEDDS is expected to present significantly higher bioavailability, bypass RMV and BRB first-pass metabolism, and reduce pill burden compared to the marketed product available for single IV infusion and frequent dosing. However, future studies should also involve in vivo animal cancer models and pharmacokinetic/pharmacodynamics studies to investigate the antitumor activity of the combined RMV + BRB bio-SNEDDS formulation. In addition, stability studies should be conducted to evaluate the stability of RMV and BRB within the formulation.

## 5. Conclusions

The current study introduces a paradigmatic case of drug repurposing which explores the potential anticancer activities of RMV and BRB. Moreover, the concept of bio-SNEDDS was explored through investigating the potential bioactivity of BSO, FSO, and ZRO on cancer cells. RMV/BRB loaded bio-SNEDDS showed acceptable visual, self-emulsification, droplet size, and zeta potential characteristics. In light of the study findings, we conclude that different concentrations of BSO and their different formulations decreased the growth of breast and lung cancer cells. Cell proliferation in breast and lung cancer cells was found to decrease in a concentration-dependent manner in response to different concentrations of BSO and its different drug-free and RMV/BRB-loaded bio-SNEDDS formulations upon 72 h exposure time.

## Figures and Tables

**Table 1:** The name and quantity of the chemical constituents present in the BSO.

S. No	Chemical Constituents	RT (min)	Area	Quality
1.	Trans Thujene	6.07	38,250,124	91
2.	Alpha pinene Dimer	6.1	8,300,185	97
3.	Alpha Thujene	6.75	3,649,660	91
4.	Beta-Pinene	6.86	7,601,772	97
5.	4-isothyocyanato-1-butene	6.97	1,825,185	86
6.	(+)-4-Carene	7.47	863,913	96
7.	Para Cymenene	7.66	99,930,937	94
8.	1-Methyl-4-(1-methyl ethyl)-1,4 cyclohexadiene	8.11	2,647,404	97
9.	Beta Terpinene	8.72	3,070,595	55
10.	1,3,4-trimethyl-3-cyclohexene-1-carboxaldehyde	9.78	1,243,371	59
11.	Terpinen-4-ol	10.08	1,670,144	94
12.	Trans-p-menthone	10.36	1,732,947	99
13.	Thymoquinone	11.19	105,710,772	78
14.	Isoborneol	11.55	613,240	99
15.	Carvacrol	11.82	8,961,583	80
16.	Longipinene	12.53	4,604,900	90
17.	Spatulenol	12.86	492,131	89
18.	Longifolene	13.46	19,892,680	76
19.	Paeonol	15.75	52,505,462	99
20.	(Z)6,(Z)9-Pentadecadien-1-ol	17.08	982,331	58
21.	Gurjunene	17.29	1,421,942	96
22.	Tetradecanoic acid	18.22	7,059,132	99
23.	1,2,3,4-tetrahydro-6-methoxy-1-salicyl-7-Isoquinolinol	18.91	1,919,745	76
24.	Methyl ester of Hexadecanoic acid	19.85	669,001	99
25.	2-Methyl-5-methoxy-4H-pyran-4-one	19.94	504,826	58
26.	cis-9-Hexadecenoic acid	20.17	662,779	99
27.	n-Hexadecanoic acid	20.82	296,221,560	95
28.	methyl ester of 9,12-Octadecadienoic acid	21.5	2,553,881	91
29.	methyl ester of 11-Octadecenoic acid	21.62	1,186,279	53
30.	3,5-dimethyl-Cyclohexanol	21.76	46,556,405	93
31.	Androstane-3,17-diol	23.37	5,925,178	90
32.	9,12-Octadecadienoic acid	23.9	14,493,017	91
33.	2-hydroxy-1ethyl ester 9,12Octadecadienoic acid	24.55	5,442,419	96
34.	Erucic acid	25.4	5,733,659	96
35.	9,17-Octadecadienal	26.37	2,041,292	96
36.	2,6,10,15,19,23-hexamethyl-2,6,10,14,18,22-Tetracosahexaene	27.49	2,119,364	98

**Table 2:** The name and quantity of the chemical constituents present in the FSO.

S. No	Chemical Constituents	RT	Area	Quality
1.	4-isothiocyanato-1-Butene	6.96	769,459	86
2.	p-cymenene	7.6	205,984	94
3.	3,3,5-trimethyl-cis-Cyclohexanol	10.13	125,880	91
4.	Heptadecane	11.35	104,463	72
5.	Decane, 2,3,7-trimethyl-	11.98	100,497	58
6.	Beta-Gurjunene	13.44	74,890	64
7.	(2-isothiocyanatoethyl)-Benzene	14.38	94,519	76
8.	Hexadecane	14.57	139,678	90
9.	2,4-bis(1,1-dimethylethyl)-Phenol	14.95	107,702	96
10.	alpha,alpha’-Dihydroxy-m-diisopropylbenzene	16.39	236,246	50
11.	n-Tetracosanol-1	17.04	81,178	72
12.	Cis-pinane	18.93	300,212	91
13.	9-Eicosyne	19.2	77,787	64
14.	Phytol	19.39	152,478	80
15.	Octacosane	19.84	146,765	83
16.	7,9-Di-tert-butyl-1-oxaspiro(4,5)deca-6,9-diene-2,8-dione	19.92	136,232	98
17.	Palmitic acid	20.35	5,948,720	99
18.	Methyl palmitate	21.07	77,500	86
19.	methyl ester of 9,12-Octadecadienoic acid	21.55	169,577	98
20.	12-Methyl-E,E-2,13-octadecadien-1-ol	21.67	185,487	53
21.	Cycloeicosane	21.74	124,790	93
22.	9,12-Octadecadienoic acid	22.11	36,041,107	99
23.	Octadecanoic acid	22.27	851,731	96
24.	13-Tetradece-11-yn-1-ol	24.39	144,138	60
25.	Methyl 9,12-heptadecadienoate	24.44	185,487	94
26.	2-octyl-Cyclopropaneoctanal	24.66	107,616	92
27.	Butyl 9,12-octadecadienoate	24.84	2,895,986	98
28.	2-Methyl-Z,Z-3,13-octadecadienol	25.04	415,487	91
29.	9,17-Octadecadienal	26.33	2,417,621	96
30.	cis,cis-7,10,-Hexadecadienal	26.79	98,610	95
31.	Squalene	27.46	1,133,348	96

**Table 3:** Robustness to dilution of the drug-loaded formulations.

	Distilled Water			0.1 N HCl			Phosphate Buffer (pH 6.8)		
	10	100	1000	10	100	1000	10	100	1000
F4 (initial)	Very Turbid	Turbid	Semi-transparent	Very Turbid	Turbid	Semi-transparent	Very Turbid	Turbid	Semi-transparent
F4 (after storage)	RPS	RPS	RPS	RPS	RPS	Stable as Semitransparent	RPS	RPS	Stable as Semitransparent
F5 (initial)	Very Turbid	Turbid	Semi-transparent	Very Turbid	Turbid	Semi-transparent	Very Turbid	Turbid	Semi-transparent
F5 (after storage)	RPS	RPS	RPS	RPS	RPS	Stable as Semi-transparent	RPS	RPS	Stable as Semi-transparent

RPS: Reversible Phase separation.

**Table 4:** Thermodynamic stability of the drug-loaded formulations.

Formulation	Centrifugation	Heat–Cool Cycles	Freeze–Thaw Cycles
F4	Reversible Phase separation	Reversible Phase separation	Reversible Phase separation
F5	Reversible Phase separation	Reversible Phase separation	Reversible Phase separation

**Table 5:** Particle size distribution data, PDI, and zeta potentials of the Bio-SNEDDS. Data are expressed as mean ± SD, n = 3.

No.	Formulation (%*w*/*w*/*w*)	Particle Size (nm)	PDI	Zeta Potential (mv)
F1	FSO	>5000	>1.00	N/A
F2	BSO	>5000	>1.00	N/A
F3	ZRO	>5000	>1.00	N/A
F4	FSO/I988/T80R (35/15/50)	151.60 ± 1.51	0.329 ± 0.059	−16.87 ± 0.55
F5	BSO/I988/T80R (35/15/50)	247.03 ± 9.18	0.441 ± 0.038	+28.73 ± 0.86

**Table 6:** Equilibrium solubility of BRB and RMV in lipid-based formulations. Data are expressed as mean ± SD, n = 3.

No.	Formulation (%*w*/*w*/*w*)	Equilibrium Solubility (mg/g)	
		BRB	RMV
F1	FSO	3.05 ± 0.09	3.17 ± 0.07
F2	BSO	4.81 ± 0.08	5.90 ± 0.10
F3	ZRO	4.22 ± 0.11	4.57 ± 0.05
F4	FSO/I988/T80R (35/15/50)	6.21 ± 0.05	8.20 ± 0.18
F5	BSO/I988/T80R (35/15/50)	7.04 ± 0.13	10.93 ± 0.07

**Table 7:** Viscosity (Cp) determination of the drug-free and drug-loaded anhydrous formulation systems and bio-SNEDDS. Data are expressed as mean ± SD, n = 3.

No.	Formulation (%*w*/*w*/*w*)	Viscosity (Cp) of Drug Free Formulations	Viscosity (Cp) of Drug Loaded Formulations
F4	FSO/I988/T80R (35/15/50)	0.717 ± 0.015	0.777 ± 0.015
F5	BSO/I988/T80R (35/15/50)	0.693 ± 0.006	0.717 ± 0.006

**Table 8:** IC 50 value of different formulations on breast, lung cancer and human fibroblasts cell line.

Name of Formulation	IC 50 Value (µg/mL)		
	Breast Cancer (MDA-MB-231) Cell Line (*)	Lung Cancer (A549) Cell Line (*)	Human Fibroblasts Cell Line
FSO	NA#	NA#	NA#
BSO	108.09 ± 6.42 ^a^	197.14 ± 2.5 ^a^	476.75 ± 38.94 ^a^
ZRO	NA#	NA#	NA#
F4-loaded RMV (single drug)	3.95 ± 0.02 ^b^	4.57 ± 0.42 ^b^	3.35 ± 0.87 ^b^
F4-loaded BRB (single drug)	2.24 ± 0.69 ^b^	3.6 ± 0.5 ^b^	4.88 ± 0.32 ^b^
F4-Drug free systems	4.14 ± 0.22 ^b^	5.78 ± 0.38 ^b^*	5.44 ± 0.24 ^b^
Pure BRB	11.1 ± 1.58 ^c^	15.85 ± 1.9 ^c^	21.92 ± 1.92 ^b^
Pure RMV	25.92 ± 3.0 ^d^	76.6 ± 4.05 ^d^	NA#
F4-systems (combined RMV + BRB)	4.2 ± 0.32 ^b^	5.06 ± 0.08 ^b^	5.04 ± 0.11 ^b^
F5-Bio-SNEDDS (combined RMV + BRB)	1.9 ± 0.19 ^b^	2.37 ± 0.22 ^b^**	3.05 ± 0.11 ^b^

NA# not available. *,** Data are expressed as mean ± SD, n = 3. Values that are superscripted with the same letter (within each column) indicate insignificant difference between the values. In the (A549) cell line column, all the values that have ^b^ superscripts are not significantly different except for F4-drug-free systems and F5-bio-SNEDDS (combined RMV + BRB) that are significantly different from each other.

**Table 9:** Bioactive lipid excipients and their combinations in the developed formulations as per lipid formulation classification systems (LFCS).

Formulation No.	FSO	BSO	ZRO	I988	T80R	Total	Formulation Type
F1	100	-	-	-	-	100	LFCS Type I (Oil only)
F2	-	100	-	-	-	100	LFCS Type I (Oil only)
F3	-	-	100	-	-	100	LFCS Type I (Oil only)
F4	35	-	-	15	50	100	LFCS Type III Systems
F5	-	35	-	15	50	100	LFCS Type III (SNEDDS)

FSO—fenugreek seed oil; BSO—black seed oil; ZRO—zanthoxylum rhetsa oil; I988—Imwitor 988; T80R—Tween 80 refined.

## Data Availability

Not applicable.
